# Improving the Prevention of Cardiovascular Disease in Primary Health Care: The Model for Prevention Study Protocol

**DOI:** 10.2196/resprot.2882

**Published:** 2014-07-09

**Authors:** Nerida Volker, Rachel C Davey, Thomas Cochrane, Lauren T Williams, Tanya Clancy

**Affiliations:** ^1^Center for Research and Action in Public HealthUniversity of CanberraCanberraAustralia; ^2^Griffith Health InstituteGriffith UniversitySouthportAustralia; ^3^ACT Medicare LocalCanberraAustralia

**Keywords:** cardiovascular disease, primary prevention, cardiovascular absolute risk, models, primary health care, mixed methods, case study

## Abstract

**Background:**

Cardiovascular disease (CVD) is the leading cause of death globally, and accounted for nearly 31% of all deaths in Australia in 2011. The primary health care sector is at the frontline for addressing CVD, however, an evidence-to-practice gap exists in CVD risk assessment and management. General practice plays a key role in CVD risk assessment and management, but this sector cannot provide ongoing lifestyle change support in isolation. Community-based lifestyle modification services and programs provided outside the general practice setting have a key role in supporting and sustaining health behavior change. Fostering linkages between the health sector and community-based lifestyle services, and creating sustainable systems that support these sectors is important.

**Objective:**

The objective of the study Model for Prevention (MoFoP) is to take a case study approach to examine a CVD risk reduction intervention in primary health care, with the aim of identifying the key elements required for an effective and sustainable approach to coordinate CVD risk reduction across the health and community sectors. These elements will be used to consider a new systems-based model for the prevention of CVD that informs future practice.

**Methods:**

The MoFoP study will use a mixed methods approach, comprising two complementary research elements: (1) a case study, and (2) a pre/post quasi-experimental design. The case study will consider the organizations and systems involved in a CVD risk reduction intervention as a single case. The pre/post experimental design will be used for HeartLink, the intervention being tested, where a single cohort of patients between 45 and 74 years of age (or between 35 and 74 years of age if Aboriginal or Torres Strait Islander) considered to be at high risk for a CVD event will be recruited through general practice, provided with enhanced usual care and additional health behavior change support. A range of quantitative and qualitative data will be collected. This will include individual health and well being data collected at baseline and again at 12 months for HeartLink participants, and systems related data collected over the period of the intervention to inform the case study.

**Results:**

The intervention is currently underway, with results expected in late 2015.

**Conclusions:**

Gaining a better understanding of CVD prevention in primary health care requires a research approach that can capture and express its complexity. The MoFoP study aims to identify the key elements for effective CVD prevention across the health and community sectors, and to develop a model to better inform policy and practice in this key health priority area for Australia.

## Introduction

### Cardiovascular Disease in Australia

Cardiovascular disease (CVD) is the leading cause of death globally, and accounted for nearly 31% of all deaths in Australia in 2011 [1]. CVD accounted for the greatest spending at 12% ($7.6 billion) of all allocated health care expenditure in 2008-2009 [2]. Addressing lifestyle risk factors (eg, physical activity, diet, and smoking) can reduce the risk of premature mortality by 66% [3]. The use of a cardiovascular absolute risk (CVAR) approach to the primary prevention of CVD, rather than the traditional single risk factor focus, is now well established and is a recommended approach to practice in Australia [4]. Despite the evidence base and demonstrated cost effectiveness of a CVAR approach, application of this method for identification and management of CVD risk is not the usual practice in Australia [5].

### Cardiovascular Disease Prevention in Primary Health Care

Primary health care is at the frontline for the delivery of services that identify, prevent, and manage CVD risk. General practice plays a key role; however, this sector alone cannot provide the ongoing behavior change support that is often required for people at elevated CVD risk. Community-based lifestyle modification services and programs provided outside the general practice setting can have an important role in supporting and sustaining health behavior change. Fostering linkages between the health sector and community-based lifestyle services has been shown to improve population prevention outcomes [6].

The advent in 2011 of Medicare Locals, Australia’s primary health care organizations, is particularly relevant to efforts to improve CVD prevention. Given their role of operationalizing the government’s primary health care improvement program, the Medicare Locals are key agents in supporting the primary health care sector by undertaking chronic disease prevention and management activities [7]. The development of the general practice workforce via incentives to employ practice nurses and funding for the community sector to deliver community-based lifestyle modification programs is also significant [8,9]. These initiatives, along with the Medicare Locals, have the potential to increase the general practice and community sector capacity to support improved CVD prevention.

### The Chronic Care Model

To support the development of chronic disease prevention and management systems, and to improve practice and enhance the collaboration between sectors, a number of models and frameworks have been used. Such models can act to communicate a vision to different stakeholders, prompt dialogue between groups, and help to define goals and objectives for activities [10]. In the area of chronic disease prevention and management, specific models have been used successfully to inform and evaluate activities. The Chronic Care Model (CCM) has been central to chronic disease policy and practice globally since the late 1990s, and has been successful in driving quality improvement in chronic disease management [11]. Studies using the CCM as an implementation framework have consistently argued that system and patient level outcomes can be achieved by the implementation of the CCM, or even just some of its elements [12].

In 2001, Glasgow et al identified the potential for the CCM to be applied to the prevention of chronic disease [13]. They noted the many activities applicable to both chronic disease prevention and management, and identified that both require a proactive and system-oriented approach. The Expanded Chronic Care Model (ECCM) developed by Barr et al [14], combines the CCM with the action areas of the Ottawa Charter, a well known framework for health promotion [15]. Both the CCM and the ECCM have been identified as having the potential to inform improved CVD prevention practice [16,17]. However, the best model to improve practice in CVD prevention in primary health care is yet to be determined.

The Model for Prevention (MoFoP) study aims to build on the CCM and related models, identifying the key elements of a new systems-based model for the prevention of CVD that informs best practice.

### The Model for Prevention Study

The MoFoP study aims to investigate a whole-of-system approach to the prevention of CVD in the primary health care setting. The MoFoP study will examine the *HeartLink* CVD risk reduction intervention in primary health care. The study will identify key elements of improved CVD prevention practice across the health and community sectors.

The aims of the MoFoP study are to: (1) test the feasibility of the *HeartLink* pilot processes including organizational readiness and resource implications for each key stakeholder group with regards to the implementation of the intervention; (2) measure the efficacy of the *HeartLink* pilot in six general practices in the Australian Capital Territory (ACT), Australia; and (3) identify the key elements of an effective model for the prevention of CVD in primary health care, and critical success factors for implementation and sustainability.

A mix of interrelating theoretical and framing approaches governing different aspects of the study have guided the study. For example, the trans-theoretical model of behavior (stages of change) informs individual behavior change [18], the normalization process theory relates to the implementation of the intervention [19], with the expanded chronic care model and complex adaptive systems approach providing overarching frameworks [14,20].

## Methods

### Study Design

#### The Two Research Elements

The MoFoP study will use a mixed methods approach to evaluate the *HeartLink* pilot, comprising two complementary research elements: (1) a case study, and (2) a one-group pre/post quasi-experimental design acting as a pilot for a proposed larger cluster randomized trial. The study design was informed by the mixed design approach used by Provost et al [21].

#### Case Study

**Figure 1:**
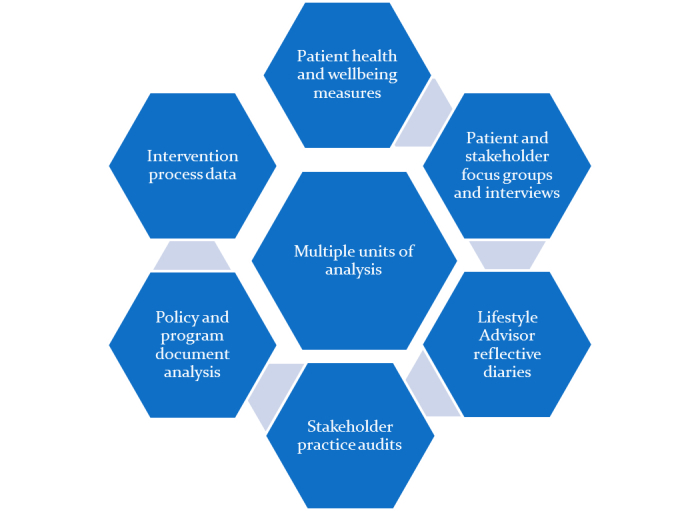
Units of analysis forming the components of the Model for Prevention (MoFoP) study.

We will use an embedded single case study design as described by Yin [22]. Yin defines a case study as an empirical inquiry that, “investigates a contemporary phenomenon within its real-life context; when the boundaries between the phenomenon and context are not clearly evident, and in which multiple sources of evidence are used”, and where case study data is collected to test preidentified theoretical propositions. The study will consider the organizations and systems involved in the *HeartLink* intervention as a single case, with a number of aspects being examined as separate units of analysis within the case. Figure 1 illustrates this idea. This strategy will use quantitative and qualitative methods, and will consider a range of measures. The outcome of this descriptive case study will be a narrative of the case.

#### One-Group Pre/Post Quasi-Experimental Design

The second component, the *HeartLink* intervention, uses a preexperimental, one group pre/post test design. A single cohort of patients 45-74 years old (or 35-74 years old if Aboriginal and/or Torres Strait Islander) considered to be at high risk for a CVD event within the next five years will be recruited through general practices, and provided with enhanced usual care and additional health behavior change support. CVAR for each patient will be determined using the Australian adaption of the Framingham based CVD risk score, with high risk being defined as a CVAR > 15% over the next 5 years [4].

The design has been informed by interventions in Australia and the United Kingdom, which also aimed to achieve CVD risk reduction through enhanced risk identification and reduction programs in primary health care [23-25]. *HeartLink* will use the ECCM as a framework to create improved prevention pathways in the primary health care sector, including providers of lifestyle modification services and programs outside the traditional health sector.

### HeartLink Practice Recruitment

*HeartLink* is set in the ACT, Australia, and is a pilot intervention in six general practices, which have been selected to broadly represent of the socioeconomic and demographic population in the region.

The practices that are recruited will be required to meet a number of inclusion criteria, including: (1) agreement that the patient has no additional payment to make from participation in the first recall visit and the 12 month follow-up visit, (2) agreement to have at least one practice nurse on staff to support the intervention, and (3) have the PEN Computer Systems Pty (Sydney, Australia) Clinical Audit Tool software.

### HeartLink Patient Sampling Strategy and Sample Size

The patient sample size for the *HeartLink* intervention was determined by an examination of similar pilot studies [25]. However, resource constraints and the desire to minimize the burden on participating practices determined the final pilot sample size of 30 patients in each of the six practices, a sample of 180 in total. Due to the variability in practice population sizes, potential losses due to poor recall response, and failure to join or complete the intervention, which could be as high as 80% of the eligible registered patients in some practices (this is estimated from pilot work on practice data download and CVAR calculation), the aim is to recall as many patients with a CVAR > 15% as possible, within the six month recruitment period. A high dropout rate is expected due to the number of steps involved in moving from patient identification, to recall and recruitment, and finally to program participation and completion. This sample size should provide estimates of key parameters, such as possible effect size for the change in CVAR for patients, so that the optimal design of a full evaluation trial can be determined (see data analysis).

### HeartLink Participant Eligibility and Recruitment

Inclusion and exclusion criteria for *HeartLink* were established. Patients needed to be 45-74 years old (35-74 years old for Aboriginal and/or Torres Strait Islander people), have a CVAR >15% over the next five years, and an absence of established CVD, diabetes, or a previous cardiovascular event to be included. Patients were excluded if they had not attended the general practice in the last two years, had a complex coexisting medical condition or impairment, were non-English speaking, or were excluded from the intervention by their general practitioner.

The patients participating in the intervention will be recruited through their usual general practice. Each of the pilot general practices uses a clinical software program to manage patient information and possesses a clinical audit tool. The clinical audit tool has a CVD risk calculator report function that generates a list of patients (from the clinical software records) meeting the CVAR inclusion criterion of associated risk level. The CVAR calculator uses age, gender, systolic blood pressure, smoking status, and serum lipids to calculate risk. A history of left ventricular hypertrophy and having diabetes are also identified.

**Figure 2:**
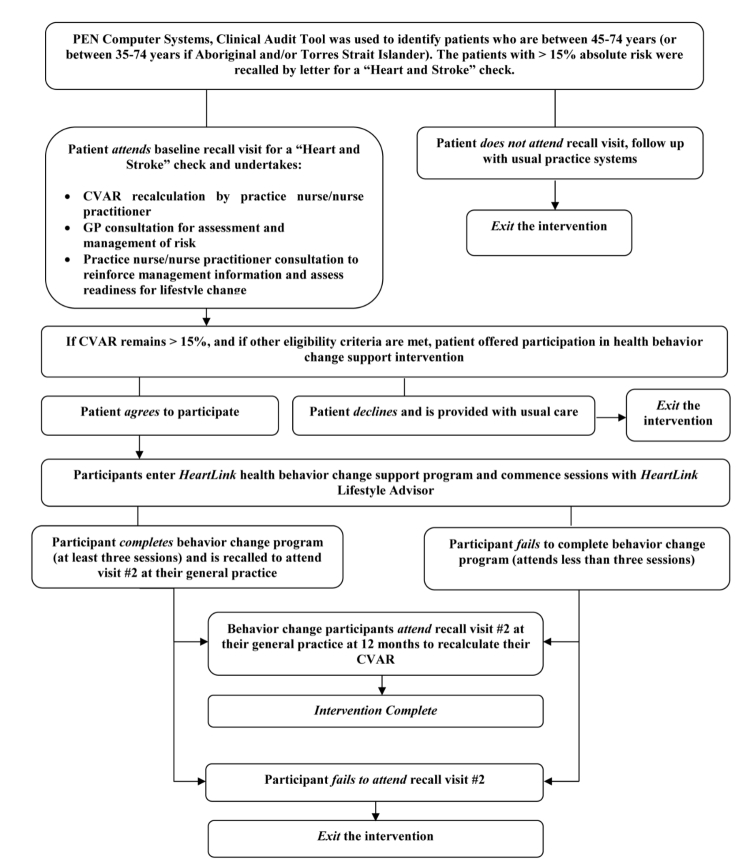
HeartLink pilot participant pathway. CVAR: cardiovascular absolute risk, GP: general practitioner.

An initial list of patients with a CVAR > 15% will be generated for each practice, and from this list random subsamples in batches of around 20 patients (the actual number depending on practice size and available resource) will be generated at scheduled intervals. As each sample is identified, the invitation letters will be posted (with up to two reminders) to invite participants to attend their practice for a “heart and stroke check”. Those attending the practice for the check will be offered participation in a health behavior change program if they are eligible. The scheduled sampling will occur over a six month period until the target sample size is achieved. Figure 2 shows the *HeartLink* participant pathway.

The University of Canberra’s Human Research Ethics Committee approved this study (Project number 11-141).

### HeartLink Intervention Description

#### Enhanced Usual Care in General Practice for Patients at High Risk of Cardiovascular Disease

The aims of the *HeartLink* pilot are to work with general practices to enhance care in relation to identification, recall, assessment, and management of patients at high risk of CVD. The intervention follows the evidence-based “5A’s” approach, with key steps integrated across the pilot design [26]. Upon recruitment, support will be provided to all of the participating general practices to audit their patient data, establish or improve data quality processes, and, where required, encourage processes to improve the levels of preventative activities such as measurement and recording of patient blood pressure. These actions are expected to maximize the patient numbers for recruitment, and will support the broader aims of the pilot to improve systems for the prevention and management of CVD in the pilot practices.

The PEN Computer Systems Pty Clinical Audit Tool CVD risk report will be used for the population of each practice, except those not attending the practice for more than two years, as it is likely that they are no longer patients of the practice or do not have a regular general practitioner (GP) at the practice. The patients identified as having a risk score of >15% on their most recently recorded clinical information will be sent a letter asking them to visit for a “heart and stroke” check [4]. The patients whose most recent cholesterol measures are greater than six months old will be sent a pathology request form along with their recall letter to encourage them to update their lipid levels before they attend their recall visit.

When patients arrive for their recall visit, they will have their CVAR recalculated by the practice nurse before a consultation with their GP.

As part of the recall visit to the general practice at baseline and 12 months, the CVD risk factor information required to calculate the absolute risk will be collected. The practice nurse in the clinical environment will measure the blood pressure, and the cholesterol level measurement (total and high-density lipoprotein cholesterol) will have been undertaken by a commercial pathology service provider upon referral from the GP prior to the visit. The smoking status, defined as smoker or nonsmoker (self-reported), will be recorded.

The GP consultation will comprise the usual care in relation to the management of the patient’s CVD risk profile, including making referrals to allied health professionals or medical specialists as required. GPs and practice nurses in participating practices will be offered professional development with regards to CVAR management guidelines, and will be provided with copies of the guidelines. After the GP consultation, the patient then returns to the practice nurse to consolidate the information provided by the GP. The patients will receive a brief intervention related to lifestyle modification and medication adherence, supported by Heart Foundation print material. The practice nurse consultation will be informed by the Health Change Australia (HCA) approach to health behavior change, and will aim to improve patient readiness for lifestyle change [27,28]. *HeartLink* uses the HCA approach across the intervention to provide patients with a consistent health behavior change paradigm, reinforced at each contact.

The eligible patients will then be offered participation in the health behavior change support program. If patients do not wish to progress to the program or are deemed by their GP to be ineligible, they will be given information on other health behavior change support options and offered ongoing usual care. The eligible patients who are initially not prepared to participate in the program will be provided with an opportunity to join the program at a later stage if they recontact their general practice within the recruitment timeframe of six months. The patients who are eligible and agree to participate in the health behavior change support program will be referred to the *HeartLink* Lifestyle Advisor linked to their practice.

#### Health Behavior Change Support Program

After referral, participants will enter into a program of health behavior change support for an average of three to six sessions over a period of up to 12 months. The completion of three sessions has been identified as sufficient time to progress key aspects of the HCA approach and achieve a moderately intensive program. These sessions will be held with Lifestyle Advisors who are fitness professionals and experienced leaders in the Heart Foundation “Heartmoves” program [29]. The Lifestyle Advisors will undertake training in the HCA approach to health change, and receive ongoing professional development sessions from HCA staff.

A short summary report will be provided back to the patient’s GP after the baseline, third, and final sessions. The advisors will connect patients with existing community resources, such as lifestyle modification programs and services to support their behavior change goals as appropriate. The advisors will also work to build relationships with local community-based providers to ensure they meet the needs of their patient cohort, and support the providers to develop systems to achieve better linkages to general practice.

#### Building an Improved Cardiovascular Disease Prevention System

Support to develop better systems for prevention of CVD will be provided to the general practice, allied health, and community sectors involved in the intervention. These activities align broadly with the elements of the ECCM. The table below provides a summary of these activities, and was based on a similar table developed for the Racial and Ethnic Approaches to Community Health program [10].

**Table 1:** Summary of *HeartLink* activities.

ECCM element	General practice activities	Community sector activities (outside general practice)
Decision support	Professional development on a CVAR assessment and management guidelines for all general practice staff.	Professional development on relevant aspects of CVAR assessment and management for allied health and lifestyle modification program providers.
	Identify decision support strategies for CVAR guidelines implementation using clinical software and complementary resources.	Identify and support the role of community-based providers in supporting the assessment and management of CVAR.
Information systems	Undertake ongoing quality activities to improve risk factor data collection and recording.	Review community and allied health information systems and identify opportunities for quality improvements related to relevant aspects of CVAR risk assessment and management.
	Develop reporting, recall, and monitoring processes for CVAR assessment of the patient population.	Identify community information systems to support community sector decision making.
Delivery system design/ reorientate health services	Develop mechanisms to improve linkages between general practices and the allied health and community sector lifestyle modification services.	Develop mechanisms to improve linkages between community sector and allied health lifestyle modification services with general practices.
	Promote the role of general practice in prevention of CVD to practice populations.	Promote the role of allied health and community sector lifestyle modification services in CVD prevention.
Self-management/ develop personal skills	Lifestyle Advisor service to provide the connection between the patient, general practice, and the community sector.	Develop and support a network of community-based lifestyle related service providers with an interest in CVD prevention.
	Deliver professional development activities to improve the health behavior change skills of general practice staff.	Deliver professional development activity to improve health behavior change skills of community sector lifestyle providers and allied health professionals.
Build healthy public policy	Map relevant national and local policy to understand aspects that support or impede the intervention approach.	Map relevant local and national policy and programs to understand aspects of policy that support and impede the intervention approach.
Create supportive environments	Promote community initiatives that are working to create supportive environments for healthy lifestyles in the general practice setting.	Develop connections with existing community initiatives that are working to create supportive environments for healthy lifestyles.
Strengthen community action	Promote the *HeartLink* intervention activities through general practice populations.	Promote the *HeartLink* intervention to the community through various media and community network channels.
	Provide opportunities to create connections between health care professionals and community organizations.	Provide opportunities to create connections between community organizations and health care professionals.

### Data Collection

#### Primary Health Outcome Measure

The primary health outcome measure for the study will be the change in the CVAR percentage score for the *HeartLink* intervention participants. A range of patient-reported health and well being outcomes will also be examined, including physical activity levels, fruit and vegetable intake, CVD medication adherence, health related quality of life, generalized self-efficacy, and intention to change key health behaviors related to CVD risk reduction.

#### Individual Health and Well Being Data

Health related data will be collected from all *HeartLink* patients using validated tools via a paper-based survey administered at baseline and at 12 months. The survey will determine: (1) Physical activity levels, the usual physical activity levels will be collected via the Active Australia survey, which has been reported as reliable and having acceptable validity within an adult Australian population [30]. (2) Fruit and vegetable intake, the number of servings of fruit and vegetables will be assessed using validated questions from the National Nutrition Survey [31]. (3) Medication adherence, the participant’s adherence to the prescribed medication (antihypertensive and lipid lowering medication) is an important factor in reducing cardiovascular risk [32]. The participant’s adherence to CVD related medication (if medication has been prescribed) will be assessed using the four-item Morisky scale for each category of medication [33]. (4) Quality of life, the health related quality of life will be assessed using the short form (SF)-12v2 Health Survey (SF12 v2). The SF12 v2 is a validated and commonly used tool that provides insight into mental and physical functioning and overall health related quality of life [34]. (5) Generalized self-efficacy measure, a validated generalized self-efficacy measure, the General Self-Efficacy Scale will be used, as self-efficacy is an important determinant of behavior change. While a number of tools have been developed to measure self-efficacy in relation to specific health promoting behaviors, a generalized self-efficacy measure was selected, given that each patient will be choosing which particular health behavior or behaviors they are ready to change [35]. And (6) Intention for lifestyle change measure, several validated questions related to key lifestyle change measures for CVD risk reduction, such as dietary changes, alcohol intake, and smoking, and based on the transtheoretical model of behavior change will be included. The lifestyle change messages are all included in the Heart Foundation printed education material provided to each intervention participant [36].

### Patient Focus Groups and Semistructured Interviews

To complement the quantitative data collection, focus group discussions or semistructured interviews will be held at the completion of the health behavior change support program. The participants of the health behavior change support program will be invited to participate in focus groups or semistructured interviews. This will allow a detailed examination of the behavior change intervention experience from the participant’s perspective. The patients who were recalled to their practice for a “heart and stroke check”, who did not attend the practice for the check, will also be invited to participate in semistructured interviews to examine the reasons for their nonattendance, and their experience of receiving the recall letter. The focus group sessions or interviews will be conducted until the saturation of themes is achieved, that is, no new major themes are detected.

### System Related Data- Case Study of HeartLink

Multiple data sources will be used to examine the complex range of system-related issues in the case study.

### Focus Groups, Semistructured Interviews, and Key Informant Forum

The focus group discussions or semistructured interviews will be conducted with key stakeholder groups over the course of the study, including with general practice staff, Lifestyle Advisors, allied health professionals, and community-based lifestyle modification program providers. These discussions will aim to identify themes in relation to the research questions, and will also provide an opportunity to engage key individuals and groups in the intervention process. Purposive sampling will be used to ensure key informants in each stakeholder group are represented. The goal will be to recruit at least eight people for each of the focus groups. The topic guides will be developed using key issues identified from the literature, including the critical success factors for systems thinking in prevention of chronic disease, and key constructs for understanding implementation of complex interventions [37,19].

The topic guides will provide an initial direction for the discussions. A forum with key policy and program informants from the primary health care sector will be conducted near the completion of the *HeartLink* intervention to gain feedback on the policy and program implications of the findings, and to provide input into the emergent CVD prevention model.

An open-ended questionnaire will be used to gather the views of the key informants.

### Primary Health Care and Chronic Disease Prevention Policy and Program Documents

Document analysis is commonly used in case study research as an additional source of evidence, and as a means of triangulation [22]. The key national and local primary health care and chronic disease prevention policy documents will be examined. The documents reviewed will include the National Primary Care Strategy, the National Primary Health Care Strategic Framework, and the National Prevention Taskforce’s National Preventative Health Strategy [38-40]. This analysis aims to provide information on the macro and meso-systems context in which the *HeartLink* intervention is occurring, provide a means of tracking change in relevant issues over the intervention period, and to verify findings or corroborate other evidence collected as part of the study [41].

### Reflective Diaries of Lifestyle Advisors

Lifestyle Advisors will keep reflective diaries for the duration of the *HeartLink* intervention. The role of the advisor is similar to that of the “boundary spanner”, as described by Etz, providing a connection between the general practice and community sector, thus offering insight into the linkage process between the sectors [42]. The advisors will be encouraged to record issues related to patients, the general practice, and their work with community lifestyle modification providers. They will be also encouraged to reflect on their own practice, and will be given a set of prompt questions encouraging them to consider barriers, enablers, opportunities, and threats for the future implementation of the intervention approach. The diaries will be reviewed regularly to ensure data quality, and will be collected at the completion of the intervention.

### Intervention Process Measures

The system’s outcomes will be further examined using a range of process measures. One of which is a brief audit to assess the degree of implementation of the CVD prevention system elements by each stakeholder group. A specific audit tool will be developed for the three key stakeholder groups: (1) general practice, (2) community-based allied health professionals, and (3) other lifestyle modification program providers based on the Assessment of Chronic Illness Care tool [43].

### Data Analysis

#### Quantitative Data Analysis

Changes in the CVAR will be reported at the total practice population level, but also analyzed by each practice. The primary analysis will be done on an Intention to Treat basis. The CVAR was chosen as the primary health outcome, as it represents an all around measure of the *HeartLink* intervention’s ability to meet its objectives.

An analysis of the quantitative data collected from the clinical data and the participant survey will be undertaken. Univariate comparisons between the pre and post intervention groups will be conducted using a chi-square test for equal proportion (or Fisher’s exact tests where numbers are small), and reported as numbers and percentages. Continuous normally distributed variables will be compared using student’s *t* tests and reported as means (95% confidence interval). Nonnormally distributed data will be compared using Wilcoxon signed rank tests.

The *HeartLink* pilot data will be used to estimate population proportions (and confidence intervals) for those at high risk of CVD in the ACT. This data will also generate planning information for a future larger study. The intercluster correlation coefficient, effect size, and standard deviation will be examined to help determine sample size calculations, cluster size, and number of clusters.

#### Qualitative Data Analysis

The thematic analysis of focus groups, semistructured interviews, reflective diaries, and key policy and program documents will be undertaken. A mixed deductive and inductive approach will be used for the analysis, which allows for initial codes to be identified from the literature, and knowledge and experience of the research team [44]. These codes will then be revised, reviewed, replaced, and added to, from the data. This process provides the inductive aspects of the analysis. Consistent with a convergent parallel mixed methods design and case study approach, the data analysis stage will include the integration of the data once the primary analysis is complete [45]. This approach will allow for the triangulation of the data to enhance validity, will help to describe the complex multi-strategy nature of *HeartLink,* and could enlighten areas of interest and relevance where empirical outcomes are not clear. This integrated analysis will be presented as the case study narrative, informing the development of the implementation model.

## Results

The *HeartLink* intervention is complete, with post intervention data collection currently underway. The results are expected in late 2015.

## Discussion

### The Model for Prevention Study

This protocol seeks to provide a detailed description of the MoFoP study, which will examine a whole-of-system CVD prevention intervention in primary health care. It will aim to identify key elements of an effective model and implementation strategies to inform better practice in this key health priority area for Australia. A recent systematic review of interventions aimed at enhancing best practice in primary health care for chronic disease management, prevention, and episodic care found that multiple and linked strategies targeting the system, practice, and community level are most likely to improve access for patients to best practice [46]. The *HeartLink* intervention, which is the focus of the MoFoP study, adopts an intersectoral and multi-level approach, which aligns with findings of this review.

Gaining a better understanding of CVD prevention in primary health care requires a research approach that can capture and express its complexity. By choosing a mixed methods research design and using the complementary case study and experimental approaches, we aim to provide a comprehensive picture of the study outcomes. While there are a number of challenges in effectively capturing the many layers in a “real life” intervention, the potential for findings to have more rapid translation into policy and practice because they present a more complete and pragmatic understanding of issues makes the approach worth pursuing.

By bringing together a range of issues of interest to contemporary primary health care, the MoFoP study provides a timely appraisal of the use of a CVAR approach to CVD prevention, the evolving role of the primary health care sector, and evidence-based approaches to supporting lifestyle change. These issues align with the current health reform agenda in Australia that commits to a reorientation of health services for a stronger preventative focus and rededication to the ideals of primary health care [38].

Finally, the MoFoP study uses existing models for improvements in chronic disease prevention as its working framework. The ECCM in particular provides a vision for an integrated, whole-of-system and intersectoral approach to CVD prevention. By developing an intervention that addresses the key elements of this model, and then undertaking a comprehensive exploration of the intervention outcomes, we hope to explore the potential to further develop the ECCM model, improving its applicability to CVD prevention in the Australian primary health care context. We also hope that an enhanced model based on the study findings will be a valuable additional outcome from the study, and will facilitate the communication and implementation of knowledge gained from this study.

### Limitations of the Study Protocol

There are a number of limitations of this study. Case study approaches have often been criticized for their lack of generalizability, and their lack of rigor. To address this, a sound research design has been developed that follows a logical process [22]. The multiple sources of data and the addition of the preexperimental component should add rigor by allowing the cross-validation of the interpretation of the study findings.

The *HeartLink* intervention itself has limitations that include the small scale of the intervention, “opt in” in the sample of practices, and the intervention occurring in one relatively small city of approximately 340,000 people. Given its pragmatic design, the study has limitations in standardizing some aspects of the intervention protocols and data collection, within busy general practices with a varied workforce and a wide range of skill sets. That acknowledged, the MoFoP study and the *HeartLink* intervention are designed to inform a larger cluster randomized controlled trial to evaluate the effectiveness of using a larger number of control and intervention practices, and to better understand the effectiveness and generalizability across the primary health care sector as a whole.

### Conclusions

The MoFoP study will examine a whole-of-system CVD prevention intervention that supports patients from risk identification through to clinical and lifestyle issues management, from general practice and into the community. By considering existing chronic disease prevention models and undertaking a holistic investigation of the *HeartLink “*case”, the MoFoP study aims to build understanding and propose new directions for this complex area of primary health care. Given the size of the problem and the current evidence to practice gap, it is an area worthy of exploration, and its outcomes should be of interest to both practitioners and policy makers.

## Abbreviations

ACT: Australian Capital Territory
CCM: Chronic Care Model
CVAR: cardiovascular absolute risk
CVD: cardiovascular disease
ECCM: Expanded Chronic Care Model
GP: general practitioner
HCA: Health Change Australia
MoFoP: model for prevention
SF: short form
